# Pragmatics Development in Deaf and Hard of Hearing Children: A Call to Action

**DOI:** 10.1542/peds.2020-0242L

**Published:** 2020-11

**Authors:** Amy Szarkowski, Alys Young, Danielle Matthews, Jareen Meinzen-Derr

**Affiliations:** a Children’s Center for Communication/Beverly School for the Deaf, Beverly, Massachusetts; b Division of Developmental Medicine, Boston Children’s Hospital, Boston, Massachusetts; c Department of Psychiatry, Harvard Medical School, Boston, Massachusetts; d Social Research with Deaf People, School of Health Sciences, University of Manchester, Manchester, United Kingdom; e Centre for Deaf Studies, University of the Witwatersrand, Johannesburg, South Africa; f Department of Psychology, University of Sheffield, Sheffield, United Kingdom; g Division of Biostatistics and Epidemiology, Cincinnati Children’s Hospital Medical Center, Cincinnati, Ohio; h Department of Pediatrics, College of Medicine, University of Cincinnati, Cincinnati, Ohio

## Abstract

Although major strides have been made in supporting the linguistic development of deaf and hard of hearing (DHH) children, a high risk of pragmatic delay persists and often goes unrecognized. Pragmatic development (the growing sensitivity to one’s communication partner when producing and comprehending language in context) is fundamental to children’s social-cognitive development and to their well-being. We review the reasons why DHH children are vulnerable to pragmatic developmental challenges and the potential to create positive change. In this call to action, we then urge (1) medical providers to recognize the need to monitor for risk of pragmatic difficulty and to refer for timely intervention (beginning in infancy), (2) allied health professionals involved in supporting DHH children to incorporate development of pragmatic abilities into their work and to foster awareness among caregivers, and (3) the research community to deepen our understanding of pragmatics in DHH children with investigations that include pragmatics and with longitudinal studies that chart the paths to positive outcomes while respecting the diversity of this population. By working together, there is substantial potential to make rapid progress in lifting developmental outcomes for DHH children.

Deaf and hard of hearing (DHH) children are a highly diverse population whose language development, whether in spoken or signed language(s), poses complex challenges for practitioners and families alike. There have been great strides in hearing technologies, the formal recognition of the status of signed languages as legitimate,^1^ and improvements across the globe in early identification of and intervention to support hearing loss.^2–5^ Collectively, these have contributed to language development trajectories and mono-, bi-, and multilingual language development unsurpassed in previous eras.^6–9^

For most children, language acquisition occurs organically; it is taken for granted that a child with typical hearing will acquire the language(s) in their developmental environment. Yet, for DHH children, there is a high risk of delays in language acquisition and development that, if not addressed, can result in a range of undesirable developmental consequences. Both early language acquisition and continued higher level language development often require direct instruction and nurturance in DHH children.^10^

For language to develop optimally, children need to learn not only linguistic forms (such as words or signs, grammatical structures, and prosodic features) but also how to deploy these linguistic resources to support effective social interaction. This is the domain of pragmatics.^11^ There is a strong association between pragmatic abilities and formal language. Indeed, many have argued that early pragmatic milestones, such as the ability to enter into joint attention and to use social vocalizations and gestures in infancy, are critical precursors of formal language.^12,13^ As children develop, pragmatic skills (including responding contingently to conversational turns, choosing felicitous expressions in context, drawing inferences about the meaning of communication partners’ comments^14^) become vital to maintaining relationships with others.

Delays and deficits in pragmatic abilities in children are present in clinical populations and are known to impact well-being.^15,16^ For example, in individuals diagnosed with autism spectrum disorder or social (pragmatic) communication disorder (formerly referred to as pragmatic language impairment), persistent pragmatic deficits are included as diagnostic criteria.^17^ Additionally, these pragmatic deficits are associated with difficulties with forming relationships,^18^ challenges with obtaining and maintaining employment,^19^ and problems across behavioral, social, and emotional areas. These populations are therefore largely recognized as requiring targeted interventions in pragmatics. Similar focused attention promoting pragmatic intervention for DHH children has yet to be established, despite ample evidence of risk for pragmatics delays and deficits in this population.

Even in the absence of a formal diagnosis, deficits in pragmatics can nonetheless have potentially lifelong consequences contributing to poor well-being^20^ and barriers among DHH children in achieving their potential as they transition to adulthood.^21,22^ Delays in pragmatic skills are common among DHH children.^23–25^ Reduced pragmatic abilities adversely impact effective communication and interaction, social and familial relations,^26,27^ and the ability to navigate contexts and environments successfully to achieve one’s goals.^28^ Yet, when recognized early, and when appropriate interventions and support are provided, reduced pragmatic abilities can be impacted positively, optimizing outcomes for DHH children.^29^ We urge the prioritization of pragmatics in DHH children for a wide range of professionals, alongside families, by issuing this call to action.

## PRAGMATIC CHALLENGES FOR DHH CHILDREN

Pragmatic delays are common among DHH children. From infancy through to adulthood, differences in developmental trajectories or delays have been observed in a range of skills, including maintaining joint attention, turn taking, topic maintenance and responding contingently to a partner during conversation, asking questions, repairing misunderstanding, and understanding jokes, deception, and sarcasm.^21,30–34^

## WHY ARE DHH CHILDREN VULNERABLE TO PRAGMATIC DEVELOPMENTAL CHALLENGES?

Conditions required for the development of good pragmatic abilities can be compromised in DHH children for 5 principal reasons.

### Reduced Formal Language

To be confident in making inferences about what someone meant by what they said, children need a secure understanding of how words and structures are used to convey meaning. For example, to understand “Mom is bringing the groceries in from the car. She doesn’t want the ice cream to melt,” one needs to infer that “she” refers to the mother, that the ice cream was part of the shopping, and so on. To construct this cognitive model, an important prerequisite is rapidly recognizing the words and grammar being used to activate relevant semantic representations. Reduced certainty on this front slows down processing and makes drawing inferences harder. Pragmatic development, therefore, tracks formal language development but is not guaranteed by it; additional skills are required. Even if a score on a standard pragmatic assessment does not indicate a threshold for concern, DHH children may still display significant delays that impact social abilities greatly.^35,36^

### Delayed Social-Cognitive Development

DHH children are also at risk for delayed social-cognitive development^37–41^ (eg, theory of mind). This understanding is critical for advanced stages of communicative development (including the understanding of sarcasm). It is considered to develop (at least in part) cumulatively from repeated and varied experience of social interaction.^42–45^ DHH children are known to have less exposure to caregivers discussing their mental states,^43^ including their own feelings, needs, and desires (ie, caregivers sharing about their own internal and mental states), than age-matched peers.^46^

### Communication Partners

All children require frequent age-appropriate interactions, with a wide variety of communicative partners, of differing degrees of linguistic richness and interactive quality, to lay the foundations for the stages of pragmatic development through childhood.^47^ DHH children often experience difficulties in their interactions with communication partners^26,48^ whether as a result of it being harder to understand or to make oneself understood, being marginalized from social opportunities,^49^ or being communicated with in ways that do not always facilitate rich conversations^50^ (eg, communication partners who, despite good intentions, share “just enough information” with a DHH child, perhaps believing that information about what is happening “in the background” is not relevant or important).

### Natural Conversations

Through everyday communication with caregivers and peers, children are socialized into appropriate language use. Social exchanges that are natural and reciprocal foster pragmatic development for all children.^51^ Because many DHH children require assistance and intensive instruction to learn language, they are routinely subject to formalized approaches to acquisition (with parents and clinicians explicitly teaching language). Yet, children can tell the difference between genuine and contrived language use from early on.^52^ In the absence of plentiful “real interaction” experiences, there is a risk that DHH children will struggle to master the natural dynamics of conversation. This perhaps explains the common clinical impression that many DHH children will sometimes miss social nuances or be rather literal in their interpretations of social interactions. Although further empirical study is sorely needed, research suggests that caregiver interaction with DHH children is more often directive^53^ and goal-orientated rather than spontaneous and playful. In conversations between DHH children and others, natural correctives within social conversation, either individually or in groups, are less likely to occur.^54^

### Incidental Learning

The opportunity to observe others socially and acquire knowledge as an onlooker, outside of direct conversation, is a common and valuable route to communicative proficiency.^55,56^ Yet, incidental learning is a challenge^57,58^ for many DHH children who may not routinely “overhear” conversation or be exposed to information in their environment through a language they can readily access, such as American Sign Language.

## RATIONALE FOR MEDICAL PROVIDERS AND ALLIED HEALTH CARE PROVIDERS TO ATTEND TO PRAGMATICS

The responsibilities of medical providers and allied health professionals supporting DHH children are well engrained:

1. ensuring that audiological assessments are completed;
2. providing education about communication opportunities (or referring to appropriate professionals who can);
3. establishing support for the family;
4. referring for hearing assistive technologies (if appropriate for a child’s hearing levels and if desired by the family and caregivers); and
5. ensuring that pathways to language and communication support (including signed language if desired) are established.^7^

However, scant attention has been given to the identification of pragmatic delay and deficit and planning for appropriate intervention. Yet, social experience is a vital component in ensuring achievement of age-appropriate cognitive and socio-emotional outcomes.^59^ Good pragmatic abilities smooth the path to a range of desirable social, emotional, educational, and economic outcomes; they also underpin positive well-being.^24^ Poor pragmatic abilities combined with challenges in communication often associated with hearing loss are detrimental to a DHH child. Health care providers are often on the front line in terms of service provision for DHH children and, in many contexts, serve as gatekeepers to services. This is a topic that does, indeed, fall within the scope of health care. Thus, we urge health care providers and allied health professionals to attend to DHH children’s pragmatic abilities.

## CALL TO ACTION

Hearing loss is among the most prevalent congenital conditions affecting children today.^60^ Given the breadth and severity of the potential impact of pragmatics on the development of DHH children, the stakes are high. There is a need and a sense of urgency for medical providers, allied health professionals, and the research community focused on DHH children to attend to pragmatics. It is the missing piece in how best to support DHH children’s development and impact on their future social skills, abilities, and well-being. We therefore issue this call to action.

## FOR MEDICAL CARE PROVIDERS

- Recognize that it is important to monitor not only the formal language development of DHH children but their pragmatic development as well. Consider whether a DHH child under your care can understand and use language effectively for social interaction.
- Appreciate the significance of pragmatic development in DHH children; reduced abilities in this area have consequences across several domains, immediate and longer term, including socio-emotional and cognitive development.
- Note when DHH children obtain passing scores on developmental screens or surveillance checklists yet do not pass screening tools that monitor language development or social communication. This should be considered a “red flag.” Further action may be necessary.
- Refer for intervention for delays in pragmatic development, whether formally evidenced or suspected. This could include early intervention (0–36 months of age) or other specialized supports (>36 months of age). Referrals should be made to monitor pragmatic development through language and communication assessments and/or developmental evaluations.

## FOR ALLIED HEALTH AND OTHER PROFESSIONALS DIRECTLY INVOLVED WITH DHH CHILDREN

- Incorporate support of pragmatic development in your regular work with DHH children.
- Enable parents and families to understand and foster the pragmatic development of their DHH children.
- Offer practical strategies to support pragmatic development in DHH children whether in the home, educational, or community environments.

## FOR THE RESEARCH COMMUNITY

- Incorporate measures of pragmatic development of DHH children in outcome and effectiveness studies with this population. Researchers are encouraged to develop a Core Outcome Set^61^ (ie, an agreed on, standardized group of outcomes, for documenting and reporting research in this domain).^62^ This can help to reduce heterogeneity across studies and facilitate meta-analysis and meta-synthesis. The COMET Initiative^63^ is the international searchable repository for Core Outcome Sets and is currently being used to track outcomes in studies of DHH children’s development and mental health.
- Consider the diversity of developmental contexts and communication partners when conducting research regarding pragmatics in DHH children. Seek to understand strengths as well as vulnerabilities among DHH children in their ability to engage socially with others.
- Prioritize prospective longitudinal studies to understand the short-, medium-, and long-term impacts of pragmatic delays and deficits in this diverse population.
- Embrace the open science agenda, to speed progress and understanding and to enhance replicability and reproducibility of rigorous research, to better understand pragmatics in DHH children.^64,65^

## ABBREVIATION

DHH: deaf and hard of hearing
